# Introducing the Study of Life and Death Education to Support the Importance of Positive Psychology: An Integrated Model of Philosophical Beliefs, Religious Faith, and Spirituality

**DOI:** 10.3389/fpsyg.2020.580186

**Published:** 2020-10-08

**Authors:** Huy P. Phan, Bing H. Ngu, Si Chi Chen, Lijuing Wu, Wei-Wen Lin, Chao-Sheng Hsu

**Affiliations:** ^1^School of Education, University of New England, Armidale, NSW, Australia; ^2^Department of Education, National Taipei University of Education, Taipei, Taiwan

**Keywords:** life education, death education, positive psychology, spiritual cultivation, optimization, wisdom, mindfulness

## Abstract

*Life education*, also known as *life and death education*, is an important subject in Taiwan with institutions (e.g., high school) offering degree programs and courses that focus on quality learning and implementation of life education. What is interesting from the perspective of Taiwanese education is that the teaching of life education also incorporates a number of Eastern-derived and conceptualized tenets, for example, Buddhist teaching and the importance of spiritual wisdom. This premise contends then that life education in Taiwan, in general, is concerned with the promotion, fulfillment, and cherishing of quality life experiences (e.g., personal contentment, happiness). One example of life education, which resonates with other spiritual beliefs and religious faiths (e.g., Hinduism), is related to spiritual cultivation and the enlightenment of life wisdom. Our own teaching of the subject, likewise, places emphasis on the goal of teaching students to seek meaningful understanding of and appreciation for three major, interrelated components of life education: *life wisdom*, *life practice*, and *life care*. It has been acknowledged, to a certain degree, that life education has made meaningful contributions, such as the creation and facilitation of a civil, vibrant society, and that many Taiwanese individuals show dignity, respect for elders, and reverence for spiritual and religious faiths. For example, aside from high-quality hospice care, many Taiwanese engage in different types of benevolent acts (e.g., providing spiritual advice to someone who is dying), where possible. Life education is a beneficial subject for teaching and learning as its theoretical understanding may help individuals cope with pathologies and negative conditions and life experiences. One negative life experience, in this case, is the ultimate fate of humankind: death. Approaching death and/or the onset of grief is something that we all have to experience. How does one approach death? It is not easy feat, and of course, grief for a loved one is personal, and some of us struggle with this. We contend that spiritual cultivation and enlightenment, arising from life education, may assist us with the topic of death (e.g., the possibility of transcendence beyond the realm of life). More importantly however, from our own teaching experiences and research development, we strongly believe and rationalize that the subject of life education could, indeed, coincide with and support *the paradigm of positive psychology* (Seligman, 1999, 2010; Seligman and Csíkszentmihályi, 2000). Forming the premise of the present conceptual analysis article, we propose that a person’s “spiritual and enlightened self,” reflecting the convergence of three major aspects of life education (i.e., philosophical reflection, enrichment of personal well-being, and spiritual cultivation), would result in the initiation and creation of a number of virtues and positive characteristics, for example, having a *positive outlook* in life, having a *perceived sense of spirituality*, showing *compassion*, *forgiveness*, etc. These virtues and quality characteristics, from our philosophical reasoning, are equivalent to those qualities that the paradigm of positive psychology advocates for. In summary, we conceptualize that the subject of life education, from the perspective of Taiwanese education, may intertwine with the paradigm of positive psychology. A person’s spiritual and enlightened self, or his/her “holistic self,” from our rationalization, is the ultimate optimal life experience that he/she may have, enabling him/her to address the gamut of life conditions and experiences. The distinctive nature of life education in this case, as a point of summary, is that it incorporates spiritual beliefs and religious faiths (e.g., Buddhist faith), encouraging a person to seek nature and divine–human relationships, as well as to contemplate and to explore the complex nature of his/her inner self. The notion of *Buddhist samsâra*, for example, as “evidence” of spirituality, entailing the endless cycle of birth, rebirth, and redeath, may provide a person with hope into the afterlife. Such esoteric discourse, we contend, is positive and optimistic, allowing individuals to discard the dividing line between life and death.

## Introduction

*Life education*, also known as *life and death education*, has gained vast interests both in terms of teaching and research development. Specifically, with reference to the case of Taiwan and its education systems, life education is taught in schools and universities, as well as applied in society in the form of educational and social programs. What is significant, however, with reference to Taiwanese education, is that the teaching and learning of life education also incorporate Eastern-derived theoretical tenets of Buddhism (Yeshe and Rinpoche, 1976; Sheng Yen, 2010), Confucianism (Yao, 2000; Havens, 2013), and spirituality (Carmody et al., 2008; Lazaridou and Pentaris, 2016). The teaching of Buddhist meditation (e.g., a focus on *enlightenment*), for example, has been incorporated to emphasize the salient nature of the study of life education—that life education is concerned with exploration of death, which is inevitable, and the fulfillment of a cherished life. How can a person overcome grief and accept that a close relative has moved on from this physical world? How does life wisdom assist a person in his daily functioning? Are immortality and the notion of transcendence beyond death a possibility? These questions, we contend, are significant, which the study of life education makes attempts to address.

Death, grief, negative outlook, and maladaptive life experience are inevitable. Overcoming these life deficiencies, obstacles, and difficulties is an important quest, which we believe the study of life education (Shi, 1987; Huang, 1993; Chen, 2017) could assist. Indeed, aside from life education, we acknowledge that the *paradigm of positive psychology* (Gilham and Seligman, 1999; Seligman, 1999; Seligman and Csíkszentmihályi, 2000) could also play a prominent role in helping to alleviate suffering, helplessness, grief, negative life conditions, etc. Our existing research inquiries into the effect of positive psychology have resulted in the development of a psychological concept that we termed as “personal resolve” (e.g., Phan et al., 2017, 2018, 2020c). Despite our emphasis on educational processes, we contend that personal resolve, briefly defined as “person’s mental resolute and “unwavering focus” to stay on task without any uncertainty or reservation to achieve optimal best” (Phan et al., 2020c, p. 450), could feature and assist individuals to overcome barriers, negative life experiences, etc.

An interesting question, though, is whether we could integrate the study of life education and the paradigm of positive psychology into a holistic framework for research development and/or implementation. To date, to our knowledge, no researchers have yet made this attempt to unify the two theoretical orientations into one coherent model. This inquiry, we contend, is significant for the purpose of cross-cultural contribution, especially when we consider the uniqueness of Eastern epistemologies and philosophical reasoning. Recently, we published an article in *Frontiers in Psychology* (Phan et al., 2020a), where we focused, in particular, on the unification of positive psychology (Gilham and Seligman, 1999; Seligman, 1999; Seligman and Csíkszentmihályi, 2000) and mindfulness from the perspective of Buddhism (Hanh, 1976; Loden, 1996; Sheng Yen, 2010). From this account, a focus on life and death education from the perspective of Taiwanese education is insightful in terms of elucidation of theoretical understanding of the relationship between the two orientations.

## Life Education: An Introduction

It is interesting to note that Taiwan places strong emphasis on the study of life education. It is a subject that is taught in school and university. Indeed, many scholars, government officials, and teachers would attest that the study of life education has played a central role in transforming Taiwanese society into what it is today—civil, democratic, robust, and stable. One clear example, in this case, is the recent COVID-19 coronavirus pandemic where Taiwan had only seven cases of death (Note: dated as of June 30, 2020) (source: https://www.worldometers.info/coronavirus/#countries). In a similar vein, high-quality service of hospice care for senior citizens in Taiwan is commendable. Some Taiwanese, for example, serve as hospice care volunteers to provide religious and spiritual advice.

Given its importance, we have devoted a complete chapter in our forthcoming book on the subject of life and death education. The history of life education in Taiwan, which we covered in detailed in this chapter, is quite interesting—for example, in part, it arose from the study of *thanatology* (Fonseca and Testoni, 2012; Doka, 2013; Meagher and Balk, 2013; Chapple et al., 2017), or death education, from the United States and other Western countries. To understand and appreciate the nature of life education, it is important for us to identify the Chinese characters of “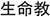,” which translate to mean “life education.” The term *life education* has been in use since 1997, when Taiwan started to promote various life education programs for secondary school teaching and learning (Ministry of Education Taiwan, 2008, 2011, 2018). Likewise, and interestingly, the Ministry of Education, Taiwan, dedicated 2001 as the Year of Life Education in acknowledgment and recognition of its significance and relevance to individuals, families, and the community. The National Taipei University of Education, where four of the authors work, has also established the Life Education and Health Promotion Institute, which serves to promote life and death education. Other institutions have similar programs and courses that promote the cultivation of life and death education.

## What Is Life Education?

Despite our brief mentioning so far, it is noteworthy to ask the question of what life education is about. We contend that this question does not have a definitive, consistent answer as the study of life education is relatively broad in scope. Our attempt to provide a balanced definition and description of life education, from historical accounts (e.g., evolution of life education with the introduction of a seminal paper by Professor Song-Yuan Huang (Huang, 1993), titled: “Death Education: A controversial subject in School Health Education”), has resulted in the following: that life education is concerned with “spiritual and personal cultivation,” via different means (e.g., formal learning of a subject titled “Spiritual Cultivation” in university) in order to elicit appreciation and meaningful understanding of life and death (Chen, 2012, 2013). In this analysis, life education is a formal process of delivery of knowledge that could, in effect, assist in the promotion, fulfillment, and cherishing of quality life experiences. From this account, it is noted that life education in Taiwan is a valuable subject matter that could educate Taiwanese citizens to appreciate and live productive life.

It is important to note, however, that life education is not simply concerned with the nature of life. Death is also a topic of discussion within the teaching of life education (Huang, 1993), hence why life education is also known as life and death education. For many Taiwanese and Asians, in general, death is a taboo topic that is often not talked about. This point is interesting as life and death are on the opposite ends of a spectrum. Life is perceived as being positive (e.g., celebration, joy), whereas, in contrast, death is negative (e.g., sorrow, grief). Any person for that matter would choose life, and not death, for studying in school and/or university. Death, of course, is inevitable and is the ultimate fate that we all have to face. Grief, sorrow, and despair, upon death of a loved one, are personal experiences that require assistance, counseling, and resolution. In this sense, life education, complementing with the teaching of Buddhism (Hanh, 1976; Loden, 1996; Sheng Yen, 2010), may assist and counsel Taiwanese to confront and face death with sense of dignity, serenity, and respect (Fu, 1993). For example, the notion of Buddhist *samsâra* may provide understanding into the possibility of “personal transcendence” beyond death itself.

An interesting question that is often asked within the context of life education is the following: *What is life?* This question, similar to that of life education, does not have a straightforward answer. Death, in contrast, is perhaps more easily defined—for example, we can define death as simply the permanent ceasing of a person or a biological organism. Life, however, is more complex and may entail different interpretations and theoretical approaches—philosophical, scientific, theological, and metaphysical speculation. From a general point of view, though, we could say that the nature of life is concerned with a person’s fulfillment of his/her purposes and goals in life, which are structured and timed for different periods in life (e.g., as part of life, the fulfillment of a goal to attend university). One Taiwanese scholar, Tsai (2008), proposes that life in itself is not a passive “pathway”; rather, there are dynamic and proactive operations and contextual influences that ultimately portray unique, differing pathways for each person. One person’s pathway, unique in its depiction, is likely to differ from other individuals’ pathways. In this discussion of Tsai’s (2008) writing, we need to consider two major points:

i. We can perceive that there are different types of life and their respective “courses” that individuals may have, which are fundamental to the appearance and interpretation of life itself. In other words, as we mentioned, a person’s life course is relatively unique, and this uniqueness, of course, may be detected and observed by us. Importantly, however, despite this uniqueness, it is poignant that different compositions within a person’s life course (e.g., a person’s employment and his/her family life) are balanced, coherent, and connected.
ii. Life course trajectory, which there are many, constitutes the total nature of a person’s life. In other words, life and life course trajectory have an inseparable relationship. Multiple life course trajectories, in this case, constitute to the totality of a person’s life. By the same token, from the above description, there are underlying operational mechanisms (e.g., a person’s mental fortitude to persist), which may then assist and facilitate a person’s successful life trajectory and/or life trajectories.

In summary, from the two aforementioned points of view, it is noted that life is made up of different life course trajectories. In a person’s lifetime, he/she may possess and manifest different life course trajectories—for example, a life trajectory as a spouse vs. a life trajectory as an employee at a local bank. We anticipate that different life course trajectories are complementary with each other, despite their uniqueness (e.g., a spouse vs. an employee). In a similar vein, of course, there are underlying operational mechanisms or principles (Tsai, 2008) that may contribute to assist and facilitate in the achievement of different life course trajectories. In the course of a person’s life as a university student, say, he/she may rely on the *process of optimization* (Phan et al., 2017, 2019a, 2020b) as an operational mechanism to assist with his/her schooling experiences. In a similar vein, another person’s life trajectory as a senior citizen may consist of his/her social relatedness to others.

### Goals and Significance of Life Education: In Brief

The preceding sections have highlighted the nature of life and briefly, likewise, the importance of death and its aftermath. Life education, as detailed, has a number of purposes that are related to both life and education. The goals of life education in this sense are unambiguous, focusing on meaningful appreciation for quality life experiences and in-depth understanding of death-related matters (e.g., the coping of grief). In our recent writing, surmising other scholars’ discussions (Huang, 1993; Chen, 2012, 2013), we purport that the main goal of life education is to *educate*, *cultivate*, and *enrich* a person’s knowledge and ability so that he/she is able to (i) continuously refine the daily practice and wisdom of life, (ii) initiate the importance of care (e.g., looking out for others), and (iii) live a meaningful life through different stages. We surmise that the goal of life education, likewise, is to empower individuals with personal belief and resolute to accept death and/or to overcome death-related matters (e.g., sorrow). For example, the teaching of Buddhism (Yeshe and Rinpoche, 1976; Sheng Yen, 2010), situated within the framework of life education places consistent emphasis on spiritual cultivation (Chen, 2001, 2009), which in this case encourages a person to seek meaningful understanding into the transcendence of oneself toward an ultimate, better self, and the transformation of the complexity of life experiences (i.e., positive and negative) into some form of unity. This emphasis of spiritual cultivation, likewise, considers the incorporation of mystical and esoteric sentiments such as a person’s quest to strive for “awakening” or enlightenment experiences, and to offer hope into the possibility of the afterlife.

The significance of life education is reflected by the objectives and subject contents that different courses and educational programs offer. In the course of a person’s life, there are three aspects for consideration in development:

i. *The wisdom of life:* It is important for a person, from birth to death, to continuously reflect on his/her acquired knowledge and experiences, which could help refine understanding into the meaning of life wisdom (e.g., “Why is it important for us, as a nation, to offer free health care?”).
ii. *The caring of life:* Life wisdom, as we briefly described, may cultivate an appropriate mindset, which could emphasize the importance of empathy, compassion, mercy, and love toward oneself and toward others. These attributes, in turn, may motivate and compel a person to show love and care for others.
iii. *The practice of life:* It is a noteworthy feat for a person to live a meaningful life with the main purpose of contemplation, refinement, and improvement. Meaningful practice of life, in this case, may consist of voluntary community service (e.g., helping out at church on Saturday).

From this understanding, it can be seen that with reference to life, there are three distinctive elements that are of significant value: wisdom, care, and practice. As we explore next, wisdom, care, and practice are inseparable and may, in fact, unite the two contrasting topics: life and death. Thus, as a point of summation, we could say that the main goal of life education is to explore the true meaning and nature of the wisdom, caring, and practice of life. In a course of study at university, for example, an educator may choose to include subject contents that focus on and/or reflect the importance of wisdom, care, and practice of life. At the same time, of course, subject contents via whole-class teaching, individual learning, and/or collaborative may also entail the study and understanding of death-related matters—for example, the coping of sorrow using faith in Buddhism as a possible means (Phan et al., 2019b, 2020a).

**FIGURE 1:**
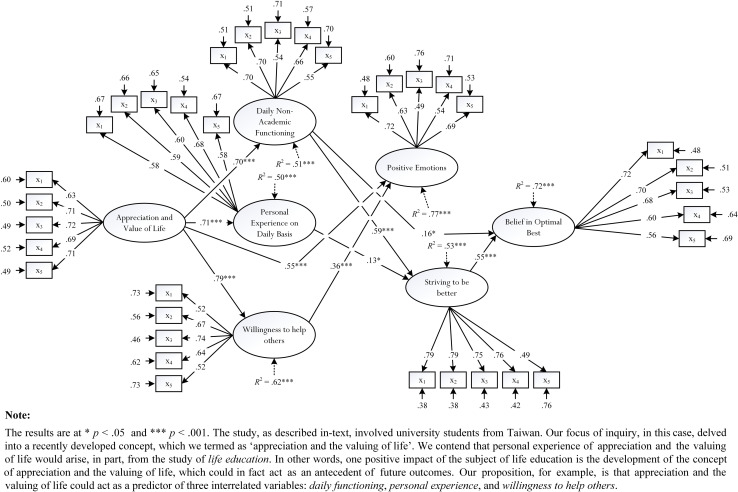
The importance of appreciation and the valuing of life.

Research into the positive effect of life education in Taiwan, over the years, has focused on different aspects of development—for example: empirical establishment, application, theorization, and conceptualization (e.g., Tsai, 2008; Huang, 2014; Chen, 2017; Tsai et al., 2018, 2019, 2020). For example, recently, we conducted an empirical study that consisted of Taiwanese undergraduate students from 12 public and private universities, where our focus of inquiry delved into the empirical validation of a concept that we developed, termed as “appreciation and the valuing of life.” For us, appreciation and the valuing of life, as a psychosocial concept, is defined as a person’s gratitude, respect, and cherishing of life and/or toward others in society (e.g., “I appreciate Bau-Yi for who she is, regardless of her poor upbringing”). Importantly, however, we conceptualized and reasoned that acquired knowledge of the subject of life education could assist in the development of the concept of appreciation and the valuing of life. Moreover, as shown in Figure 1, we postulate that personal experience of appreciation and the valuing of life could act as a potent antecedent of future outcomes. Using Likert-scale measures and the statistical technique of structural equation modeling (Loehlin, 2004; Kline, 2011), we found evidence to substantiate our hypothesized *a priori* model—for example, appreciation and the valuing of life positively predicted the concepts of daily functioning (β = 0.70, *p* < 0.001), personal experience (β = 0.71, *p* < 0.001), willingness to help others in society (β = 0.79, *p* < 0.001), and happiness (i.e., positive emotions) (β = 0.55, *p* < 0.001). This study overall, we contend, is significant as it underlines the potency of the study of life education—that meaningful understanding life education, in this case, could yield in the development of perception and personal experience of appreciation and the valuing of life.

Aside from empirical research, we have noted that many scholars, educators, government officials, etc. have also made concerted efforts to advance the study of life education. One interesting line of inquiry has involved advancement into the nexus between research and application. This emphasis, in this case, focuses on the successful transformation of theoretical tenets of life education into practice. For example, in 1990, a Buddhist Master, Master Xiao Yun, used “Enlightenment Education” as a philosophical foundation to establish Huafan University, currently located in New Taipei City, Taiwan. Master Yun advocated the use of *humanistic education* (e.g., the study of self-actualization) to enrich a person’s mindset. Master Xiao Yun, in particular, believed in the integration of both humanities and technological advances and that, likewise, there was a need to acknowledge and recognize the importance of compassion and wisdom. To promote this thinking, Huafan University offered a unit, titled “Enlightenment Wisdom and Life,” which was compulsory for enrolment. This enlightenment education is based on the theoretical premise of enlightenment, taking into consideration both Chinese culture and Buddhist teaching. Specifically, reflecting our previous mentioning, this unit incorporates contemporary pedagogical practices and places emphasis on the integration of both humanities and technological advances, as well as the promotion of compassion and wisdom (Shi, 1987). It was hoped at the time that enlightenment education, focusing on the cultivation of compassion and life wisdom (e.g., caring for another person), would bring peace and happiness to Taiwanese society.

Other institutions in Taiwan, similar to Huafan University, have also made attempts to highlight and promote the importance of life education. For example, from our observations and professional experiences, we note that there are pastoral care programs, courses and degree programs, extracurricular and social activities, and campus events that place emphasis on religious and spiritual cultivation, the enrichment of personal well-being, and the proactive caring for others (Chen, 2017). One notable goal from these positive initiatives is to educate and to encourage students to practice care, love, compassion, and life wisdom (e.g., looking out for friend in time of needs). At Huafan University, even to this day, there are weekly classes on Buddhist meditation practice and mindfulness that are intended to cultivate spirituality and beliefs in religious sentiments (e.g., the seeking of understanding of transcendence).

## The Paradigm of Positive Psychology

The main premise of the present conceptual article is to consider the possibility that life education, in general, could coincide with and/or support and substantiate the paradigm of positive psychology (Gilham and Seligman, 1999; Seligman, 1999; Seligman and Csíkszentmihályi, 2000). As we briefly mentioned in the preceding sections, we recently published a conceptual-analysis article, titled “Advancing the Study of Positive Psychology: The Use of a Multifaceted Structure of Mindfulness for Development,” which explored the interrelationship between Buddhist mindfulness (Hanh, 1976; Loden, 1996; Sheng Yen, 2010) and positive psychology. This conceptual analysis is poignant as it acknowledges the possibility that both Western and Eastern epistemologies and philosophical rationales (e.g., psychological thoughts vs. spirituality) could combine into a holistic framework, which would then provide detailed information about the proactivity of human agency (Bandura, 1986, 1997). The advent of technologies and globalization has encouraged social dialogues and, importantly, the sharing of knowledge, ideas, opinions, and viewpoints across cultures. Our cross-institutional research collaborations over the past 10 years, for example, have resulted in our propagation of meaningful and interesting cross-cultural discussions of topical themes, such as subjective well-being, optimal best, and personal fulfillment. How does life education, from the perspective of Taiwanese education, fit in with the teaching of positive psychology? An alternative question that we could inquire, likewise, is whether and/or to what extent positive psychology would fit in and support the teaching of life education. This attempt to integrate the two theoretical frameworks is innovative as it would add credence to the prominence of positive psychology as a driver of life’s proactivity and, by the same token, the promotion of the teaching of life education in school, college, and society.

### Positive Psychology: An Overview

Positive psychology, in brief, explores life conditions and experiences both in terms of negativities (e.g., the remedy of pathologies and maladaptive functioning) and positivities (e.g., the encouragement and promotion of enriched life conditions) (Gable and Haidt, 2005). This theorization of positive psychology (Gable and Haidt, 2005) largely arises from the work of Seligman, Csikszentmihalyi, and Peterson, who seek to understand the psychological well-being and optimal functioning of people (Quick, 2008). From the literature, Seligman and Csikszentmihalyi (Seligman and Csíkszentmihályi, 2000) have been credited with coining the term “positive psychology.” According to Sheldon et al. (2000), positive psychology is defined as:

“the scientific study of optimal human functioning. It aims to discover and promote the factors that allow individuals and communities to thrive. The positive psychology movement represents a new commitment on the part of research psychologists to focus attention upon the resources of psychological health, thereby going beyond prior emphases upon disease and disorder”.

This definition, as reflected in Pawelski’s (2016) comprehensive review of this topic, suggests that positive psychology incorporates and emphasizes personal characteristics, such as *internal drive*, *character building*, *human strength*, and *family and civic virtue*. From this emphasis, the study of positive psychology may entail the “building of the most positive qualities of an individual” (Seligman, 1999, p. 559) and “on building of what makes life most worth living” (Seligman, 1999, p. 562).

Seligman and Csíkszentmihályi’s (2000) published work, likewise, emphasizes the science of positive psychology may exist on three levels—subjective, individually, and institutional: “the field of positive psychology at the subjective level is about valued subjective experiences: well-being, contentment, and satisfaction (in the past); hope and optimism (for the future); and flow and happiness (in the present). At the individual level, it is about positive individual traits: the capacity for love and vocation, courage, interpersonal skill, aesthetic sensibility, perseverance, forgiveness, originality, future mindedness, spirituality, high talent, and wisdom. At the group level, it is about the civic virtues and the institutions that move individuals toward better citizenship: responsibility, nurturance, altruism, civility, moderation, tolerance, and work ethic” (p. 5).

Interestingly, Ancient Greek philosophers refer to the concept of “eudaimonia,” which is translated to connote good spirit, happiness, and a state of flourishing. Psychologists in early as the 1950s, likewise, recognized the need to examine the virtue of strength-based approaches to prevent and treat a person’s mental illness. In the area of human motivation (Franken, 2007), likewise, Maslow’s (1954a, 1962) *humanistic theory* describes the importance of self-fulfillment of inner psychological needs. Maslow (1954b) argued that the science of psychology has been far more successful on addressing the negatives than improving the positives. It has revealed man’s and woman’s shortcomings, his/her illness, and his/her sins, but little about his/her potentialities, his/her virtues, his/her achievable aspirations, or his/her full psychological height (Maslow, 1954b, p. 354).

Indeed, positive psychology has been and is difficult to define because of its nature and broad scope of psychological domains (Donaldson et al., 2015). Over the years, of course, there have been different theories proposed to explain and/or to reflect the tenets of positive psychology—for example, Phan et al.’s (2017) *Framework of Achievement Bests*, Seligman’s (2010) *PERMA* model (i.e., Positive Emotions, Engagement, Relationship, Meaning and Accomplishment), Keyes’s (2002) *Continuum of Psychological Well-Being*, and Peterson and Seligman’s (2004) *Character Strengths and Virtues Framework*. In their seminal writing, Seligman and Csíkszentmihályi (2000) defined positive psychology as “the combination of valued subjective experiences, which could contribute to the optimal experience of well-being.” This testament, based on our analysis, may incorporate reflection of a person’s past (e.g., achievement), as well as his/her hope and optimism for the future (e.g., positive emotions), and flow and happiness in the present moment (e.g., engagement and meaning) (Phan et al., 2020a). This personal experience, indeed, attests to a continuation of time: past, present, and future. At an individual level, it is operationalized through positive individual traits such as the capacity for love, vocation, courage, interpersonal skill, aesthetic sensibility, perseverance, forgiveness, originality, future mindedness, spirituality, high talent, and wisdom. At the community or the organization level, in contrast, positive psychology is concerned with civic virtues and the goal of institutions to move individuals toward better citizenship, responsibility, nurturance, altruism, civility, moderation, tolerance, and work ethic (Seligman and Csíkszentmihályi, 2000).

### Significance of Positive Psychology: Striving to Achieve Optimal Best

As noted from the preceding section, positive psychology may act to negate pathologies and maladaptive states of functioning (e.g., continuing failures in mathematics learning) and, in contrast, to also promote positive life conditions and experiences (e.g., enjoyment in seeking mastery in music composition). The *theory of optimization* (e.g., Fraillon, 2004; Phan et al., Phan et al., 2017, 2019a, 2020b), recently developed to coincide with the paradigm of positive psychology, interestingly places more emphasis on positive life experiences and adaptive outcomes—for example, the achievement of optimal best (Liem et al., 2012; Phan et al., 2016). This focus of development (i.e., a focus on the achievement of optimal functioning) is beneficial and insightful as it seeks to promote the importance of positive life conditions and different types of adaptive outcomes. It is a central feat of human agency that individuals in society strive to achieve their optimal bests and flourish in life. In terms of schooling, for example, what is it that could encourage a student to strife for optimal best in gymnastic? In a similar vein, in a non-academic sense, what could we do to cultivate optimal health?

Optimal best, also known as optimal functioning, is an important hallmark of positive psychology (Seligman and Csíkszentmihályi, 2000; Fraillon, 2004; Phan et al., 2019a) and, by the same token, is an antithesis of pessimism, procrastination, a state of demotivation, and suboptimal functioning. Optimal best, as the term connotes, is concerned with successful accomplishment and/or fulfillment of a state of functioning (e.g., cognitive functioning) that, in this case, reflects the maximization of a person’s capability (Fraillon, 2004; Phan et al., 2019a). In terms of diversity, for example, achievement of optimal best may involve the following:

• Academic learning, for example, a student’s optimal cognitive functioning in essay composition, where he is able to write a 5,000-word essay and subsequently receiving an A^+^ grade.
• Personal well-being in a workplace environment, for example, a bank employee’s optimal state of resilience, personal resolve, and motivation to overcome difficulties and achieving exceptional KPIs.
• Health functioning on a daily basis, for example, a senior citizen’s optimal state of health despite her recent temporary illness from the COVID-19 pandemic.
• Professional sports performance (e.g., European football), for example, a football player’s optimal physical and creative ability to score 50 goals in the 2020/2021 season.

The above examples emphasize the general nature of optimal best for different life contexts. Optimal best, which Fraillon (2004) also terms as “notional best,” is a point of reference by which a person strives to achieve. “What is my optimal best?” indeed is a phrase that one may commonly use as a source of aspiration and motivation to succeed. In their recent article, Phan et al. (2019a) provided a comprehensive overview and conceptual analysis of this concept of optimal best. According to the authors, determination of optimal best in a subject matter (e.g., a football player’s optimal physical and creative ability), denoted as “L_2_,” requires some form of “benchmarking” or referencing from a current level of best practice, denoted as “L_1_.” Interestingly, from their conceptualization, Phan et al. (2019a) argued that the difference between L_1_ and L_2_, denoted as Δ_(L__1_–_L__2__)_, would represent and define a person’s *state of flourishing*. From this account, we contend that a person’s experience of flourishing, Δ_(L__1_–_L__2__)_, entails some positive, enriched quantitative and/or qualitative change. Importantly, however, Phan and his colleagues’ research work of optimal best is seminal and innovative for their emphasis on the process of optimization—that is, what is it that governs and causes a person to achieve optimal best?

Positive psychology does not simply focus on optimal functioning, nor does it entail the masking of negative life conditions and experiences (Pawelski, 2016). Indeed, helplessness, sorrow, depression, continuing failures, and despair are some notable pathologies and negative life conditions, which we contend are noteworthy for consideration and addressing. In brief, the taboo subject of death itself is something that we all have to confront. It is the ultimate fate of humankind: the ceasing of life itself. In life, there are many personal situations, circumstances, events, experiences, etc. that are ongoing and/or repetitive, allowing us to recall and inform others—for example, “…it was like this for me when the COVID-19 coronavirus pandemic happened…,” and “…I really enjoyed the music concert the other day….” Death, however, is not an experience, situation, events, etc. that we can recall and repeat to someone (e.g., “…. for me, death was like …”). Interestingly, there is an article by Jennie Dear (September 9, 2016) published in *The Atlantic* (titled “What It Feels Like to Die”) that seeks to clarify the question of what it feels like to die. By all accounts, Dr. James Hallenbeck, a palliative care specialist at Stanford University, compares dying to blackholes—“We can see the effect of black holes, but it is extremely difficult, if not impossible, to look inside them. They exert an increasingly strong gravitational pull the closer one gets to them. As one passes the ‘event horizon,’ apparently the laws of physics begin to change” (source: https://www.theatlantic.com/health/archive/2016/09/what-it-feels-like-to-die/499319/).

Dear’s (September 9, 2016) article and personal account of death is interesting as it makes attempts to understand death. Of course, the article does not completely elucidate the complex nature of death in terms of the emergence of onset experience (e.g., one’s onset experience as he/she approaches death), grief, suffering, etc. Despite this caveat, the article does, in part, support the use of positive psychology, as a form of remedy, to address death and other related negative aspects of life (e.g., a person’s experience of trauma). Moreover, as an interesting premise from Dear’s (September 9, 2016) article, we contend that the subject of life education in itself could coincide with the paradigm of positive psychology (Seligman, 1999, 2010; Seligman and Csíkszentmihályi, 2000) and serve as an important remedy. That life education, in this sense, could play a prominent role in helping to alleviate negative emotions, feelings, and experiences and, by contrast, improve and enhance positive life characteristics (e.g., a state of personal resolve). From this account, we posit that life education (Chen, 2012, 2013; Huang, 2014), as a subject, could act as an informational source, which then would help to facilitate in the achievement of optimal best, academically and non-academically. Let us now consider this possibility in detail in terms of the focus of theoretical orientation.

## Life Education and Positive Psychology

By all accounts, we acknowledge that life education is not the only subject and/or theory that could coincide with the paradigm of positive psychology (Seligman, 1999, 2010; Seligman and Csíkszentmihályi, 2000) and/or that it is the only framework, which could facilitate a person’s achievement of optimal best. Our examination of the literature, for example, has indicated that there are a number of notable theories that may successfully explain the achievement of optimal best: academic buoyancy (e.g., Martin et al., 2013; Collie et al., 2015), personal thriving (e.g., Su et al., 2014; Wiese et al., 2018), and academic striving (e.g., Phan and Ngu, 2020; Phan et al., 2020c). What is unique, though, from our proposition, is that the study of life education may delve into the premise of philosophical psychology, spiritual psychology, and religious psychology. Its scope, as we indicated earlier on, is more broader than just the teaching of theories pertaining to the different stages of human development (e.g., stage of cognitive development) (Inhelder and Piaget, 1955/1958; Erikson, 1968).

Life education from the perspective of Taiwanese education is quite unique in terms of its theoretical premise, which we contend may coincide with other religious faiths, cultural practices and values, and philosophical beliefs, for example, Christianity (Davis, 2004; Knitter, 2009; Van der Merwe, 2018), Hinduism (Warrier, 2006; Srivastava and Barmola, 2013; Goswami, 2014), and Islam (Nasr, 1987/2008; Bonab et al., 2013; Marzband et al., 2017). In this analysis, as a point of prominence, life education’s focus is more philosophical, spiritual, and personal, delving into a person’s inner self and his/her relationship with nature, others in society, and some form of “divine being” (e.g., Buddha). At its core, perhaps, is the fact that the teaching of life education makes a concerted attempt to promote and facilitate the enrichment of personal well-being via means of what we term as “divine–human relationships” (Bonab et al., 2013). Enrichment of subjective well-being (e.g., a person’s experience of optimal health well-being) is positive and, in this case, reflects one of the theoretical tenets of positive psychology (Seligman, 1999, 2010; Seligman and Csíkszentmihályi, 2000), namely, to encourage and promote positive life experiences and conditions (i.e., in this case, to encourage and promote optimal health well-being). Of particular interest, from our point of view, is that life education shares a point of commonality with other religious faiths and philosophical beliefs (e.g., Saksena, 1970; Jones, 2004; Kourie and Ruthenberg, 2008; Marzband et al., 2016) in terms of emphasis in acknowledgment of the importance of nature and its intimate association with life, inclination and human attachment to some form of divine being (e.g., the importance of God), and the seeking of spiritual cultivation and connectedness between personal and transpersonal realms (Nasr, 1987/2008; Bonab et al., 2013). This theoretical contention, interestingly, suggests that life education espouses the importance in unity of the spirit, the mind, and the body in both worldly and non-worldly esoteric contexts.

It is evident that there is consensus among researchers’ conceptualizations and established findings (e.g., Saksena, 1970; Jones, 2004; Koenig, 2012; Marzband et al., 2016; Wagani and Colucci, 2018; Villani et al., 2019), which showcase the positive impact of religious faith, spiritual cultivation, and personal enlightenment toward the optimization of one’s personal well-being. Bonab et al.’s (2013) theoretical overview of Islamic spirituality, for example, is interesting as it highlights the relationship between personal religious commitment and mental health and coping ability (Miller and Thoresen, 1999). In this analysis, a person’s perceived positive relation with God is likely to enhance his/her coping and mental health condition (Galanter et al., 1979; Pargament, 1997; Belavich and Pargament, 2002). Testament of appreciation of spirituality, likewise, has also been found to make a profound impact on a person’s well-being. In a recent study in North India, interestingly, Wagani and Colucci (2018) explored an important “negative” aspect of positive psychology with reference to Hindu faith—in this case, the remedy and prevention of pathologies and maladaptive experiences. The authors found that spirituality (e.g., defined as “the improvement and knowledge of oneself. Spirituality… defined as a way to know oneself, the inner self, or the soul”: Wagani and Colucci, 2018, p. 5) positively impacted on a student’s well-being and, more importantly, served as a protective measure against suicidal tendency.

As we have acknowledged earlier, the subject of life education is not new to Taiwan and has, in fact, been credited elsewhere in terms of theoretical development. What is novel, though, is that Taiwan has placed strong emphasis on the application of life education theories into practice. How can life education assist individuals to appreciate their sense of self-worth? How can life education negate a person’s perception of life dissatisfaction? How does life education complement a person’s emphasis on a need to have financial wealth? These questions are authentic and have life-related relevance, emphasizing the importance of theoretical understanding into the multiple purposes of life, personal reflection, and philosophical reasoning, all of which account for the goals of life education. That life education, specific to the case of Taiwan, is concerned with a focus on enrichment of personal well-being, appreciation for life and the fulfillment of life qualities, and the development of coping mechanisms to deal with life matters.

Our quest is to consider a coinciding support for positive psychology from the study of life education. How does the subject of life education, from the perspective of Taiwanese education, support and/or coincide with the theoretical tenets of positive psychology? This consideration acknowledges, from our viewpoint, the perspective and understanding that life education, in general, is positive and/or that it entails positive life characteristics for development (e.g., emphasizes the importance of positive emotional well-being). On this point, we concur and strongly believe that life education, in terms of its proposed theoretical tenets (e.g., a focus on the development of life wisdom, which may consist understanding of spiritual cultivation), is a positive subject that may soundly support the paradigm of positive psychology. This testament, indeed, is the hallmark of this conceptual analysis article.

Let us now explore three major components of life education, which we consider as being prevalent to supporting our seminal postulation—that the subject of life education is closely aligned with and in support of the paradigm of positive psychology (Seligman, 1999, 2010; Seligman and Csíkszentmihályi, 2000). Our proposition, drawn from professional teaching practices and existing theoretical and research development, posits that *life wisdom* (i.e., acquired knowledge pertaining to the importance of spirituality), *personal daily engagement of life practices* (e.g., engagement in meditation), and *one’s willingness to show care for others in society*, in general, produce a number of positive life qualities, for example, a person’s positive outlook in life. In a similar vein too, from our point of view, Taiwanese life education studies (Chen, 2012, 2013; Huang, 2014) also facilitate and enhance the following:

***Philosophical Reflection:*** Personal reflection, we contend, encourages a person to internalize and to reflect on daily happenings in a philosophical manner in order to attain meaningful understanding. The main premise, in this case, is for a person to philosophically reflect and reason why things in life happen and why they are the way they are. Buddhist meditation, resulting in calmness and serenity of the mind (Loden, 1996; Phan et al., 2020a) may, in this sense, enable the person to reflect from “within the mind” and to provide sound philosophical reasoning. As an example, consider the case of social stigma and its subsequent effect (Major and O’Brien, 2005; Frost, 2011) that an adolescent is experiencing. Philosophical reflection, in this analysis, would encourage a person to seek understanding from all sides, not necessarily for the purpose of resolution for this social mishap. This act, in turn, formulates evidence of life wisdom, which a person may refer to and use for other contexts.

***Spiritual Cultivation:*** Spiritual cultivation, also known as the cultivation of spiritual mind, seeks to enlighten a person in a religious and/or spiritual sense. Specifically, with reference to the incorporation of Buddhist teaching (Yeshe and Rinpoche, 1976; Sheng Yen, 2010), Taiwanese scholars and students alike believe that spiritual cultivation, via means of acquired knowledge (e.g., Buddhist scripture) and meditation practice, would enable a person to attain meaningful understanding and appreciation of esoteric matters, such as the possibility of transcendence beyond the realm of death itself, unexplained phenomena of this physical world, and the true meaning of satori (Phan and Ngu, 2019; Phan et al., 2020a), that is, the achievement of perfection and/or tranquility. Spiritual cultivation, indeed, is a form of teaching, both formal and informal, which may serve to enlighten a person’s view and mindset of the world in a positive manner. For example, compassion, love, and willingness to forgive are all evidence of the success of spiritual cultivation.

***Enrichment of Personal Well-Being:*** Enrichment of personal well-being reflects the nature of life care in which a person may show love, care, and compassion for oneself and for others in the community. Enrichment of personal well-being may reflect a person’s mindset, as well as his/her physical being. Importantly, however, enriched personal well-being may espouse the development and acquired experience of a “spiritual self”—in this case, the perception in experience of calmness, serenity, peace, and harmony with reference to the surrounding. Moreover, research development and the study of life education (e.g., Chen, 2001, 2009; Chen, 2012) in Taiwan posit that spiritual cultivation (e.g., the teaching of satori) could assist and encourage individuals to show willingness in care, love, and wisdom for others’ well-being. Taiwan is relatively advanced in terms of quality services of hospice care to senior citizens. One of the authors of this article, for example, works as a volunteer on weekends and afterhours to look after senior citizens. His personal account, as he describes, entails spiritual advice, which may consist of Buddhist chanting and reading of Buddhist scriptures.

From our rationale, we contend that philosophical reflection, spiritual cultivation and enlightenment, and a person’s enriched well-being are positive experiences and characteristics that may arise from the study of life education. By all accounts, we acknowledge that this viewpoint and rationalization may resonate elsewhere with other religious faiths, teaching subjects, cultural practices, etc. Indeed, as attested from the extensive literature, religious faiths such as Christianity (Davis, 2004; Knitter, 2009; Van der Merwe, 2018), Hinduism (Warrier, 2006; Srivastava and Barmola, 2013; Goswami, 2014), and Islam (Nasr, 1987/2008; Bonab et al., 2013; Marzband et al., 2017) also explore the importance of spiritual cultivation and life enlightenment. For example, as we briefly referenced, Hindu faith places emphasis on a need for a person to “know and live in the highest self, the divine, the all-embracing unity, and to raise life in all its parts to the divinest possible values” (Goswami, 2014, p. 242). What is poignant, however, is that Taiwanese society and education systems place self-awareness and prominence on these elements, all of which are quality characteristics that coincide with positive psychology (Seligman, 1999, 2010; Seligman and Csíkszentmihályi, 2000).

### A Proposition for Consideration

**FIGURE 2:**
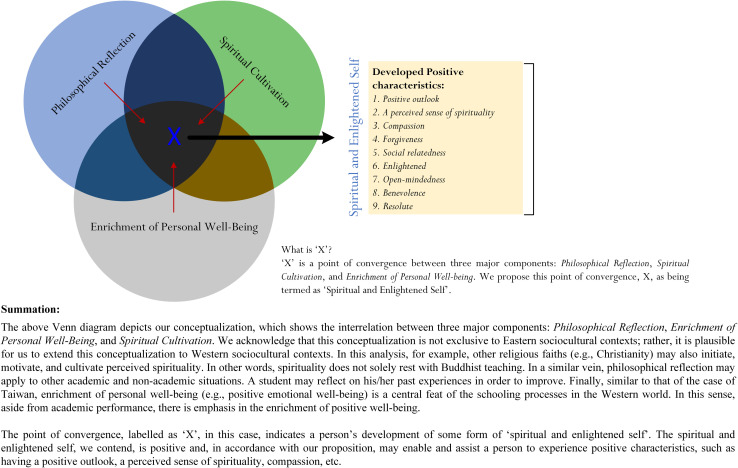
The development of spiritual wisdom and life enlightenment.

Drawing from the preceding section, our proposition considers the extent to which life education would yield quality life characteristics that, in turn, support the paradigm of positive psychology (Seligman, 1999, 2010; Seligman and Csíkszentmihályi, 2000). Positive psychology, in a general sense, entails the promotion of optimal best, academically [e.g., optimal cognitive achievement (e.g., mastery) in Algebra] and non-academically (e.g., optimal emotional well-being despite one’s obstacle). We rationalize that, in this instance, spiritual cultivation and enlightenment, philosophical reflection, and enriched well-being, all of which are positive qualities, would naturally converge, resulting in the development of life-relevant characteristics, which are shown in Figure 2 and summarized in Table 1.

**TABLE 1:** Characteristics of spiritual wisdom and life enlightenment.

**Characteristics**	**Definition**
1. Positive outlook	A person’s non-judgmental, positive outlook about life—for example, that everything in life is aesthetic regardless of negative life conditions, obstacles, difficulties (e.g., racism)
2. Perceived sense of spirituality	Acquired knowledge and manifestation of spiritual belief—for example, a person’s striving to appreciate and/or to engage in the practice of tranquility.
3. Compassion	A person’s willingness to show love, empathy, and care for others in society, regardless of their ethnicity, race, social standing, political affiliation, etc.
4. Forgiveness	A person’s willingness to be non-judgmental and to forgive others for their deeds, regardless of subsequent effects. Forgiveness, in this sense, reflects a person’s magnanimous state
5. Social relatedness	Social relatedness is more than just evidence of proactive social interactions between individuals. It is also about empathetic thoughts and acts, such as offering friendship to a person who is in need
6. Enlightened	The word “enlightened” arises from personal understanding and/or, perhaps, experience of enlightenment, or nirvana. This emphasis identifies the beatitude of life, both in the physical sense and the esoteric sense
7. Open-mindedness	A person’s understanding, acknowledgment, and acceptance that anything in this physical world is possible. This description, in particular, reflects a person’s state of inquisitiveness to understand about the world
8. Benevolence	A person’s disposition to engage in charitable acts and to show kindness and good will to others in the community. Benevolence, in this case, may also reflect sacrifice and willingness to go beyond of what is expected
9. Resolute	Personal resolute, a concept recently developed, emphasizes a person’s unwaivered focus, concentration, and mental fortitude, which may then account for his/her state of decisiveness in a particular context.

Our rationalization for the support of positive psychology (Seligman, 1999, 2010; Seligman and Csíkszentmihályi, 2000) from the study of life education (e.g., Chen, 2001, 2009; Chen, 2012) is shown in Figure 2. Foremost from Figure 2 is our conceptualization, shown as a Venn diagram, which depicts a point of convergence (i.e., denoted as ‘X”) between three described components—spiritual cultivation and enlightenment, philosophical reflection, and enriched well-being. In particular, we propose that life education would create a specific form of knowledge, understanding, and personal experience that we term as “spiritual and enlightened self.” A person’s spiritual and enlightened self is religious and spiritual and, from our proposition, may emphasize his/her intimate connectedness with some form of divine being and/or divinity. This point about spirituality and enlightenment, in a general sense, is not novel and has been noted to resonate with other religious faiths. For example, aside from Islam faith, Hindu faith, etc., Christian spirituality also recognizes the importance of a “dynamic divine–human dialogue—between the divine and the spiritual person” (Van der Merwe, 2018, p. 9). What is of consistency, perhaps, is that a perceived sense of spirituality could give rise to a person’s “eureka moment” of awakening, or enlightenment (Schneiders, 1986; Knitter, 2009), resulting in his/her experience of connectedness with nature, people, and divinity (Van der Merwe, 2018).

What do the characteristics, or “virtues,” in Table 1, as we conceptualize, actually represent? Our rationale, in this analysis, posits the following: that the study of life education, focusing on the teaching of philosophical reflection, the cultivation of spirituality, and the enrichment of personal well-being would give rise to the “creation” of a holistic entity—a person’s spiritual and enlightened self. When a person experiences spirituality and feels enlightened or awakened) (i.e., his/her holistic self), he/she is likely to exhibit different types of life characteristics and virtues such as having a positive outlook about life, being compassionate and showing forgiveness, etc. By all accounts, these positive life characteristics (e.g., a positive outlook) are comparable with those established elsewhere, especially in relation to other theoretical perspectives, cultural viewpoints, religious faiths, and customary practices. For example, our description of the positive characteristic of “positive outlook in life” (i.e., a person’s inclination to be positive about life, regardless of his/her current situations, etc.) has been referenced in existing research development pertaining to the subject of *future time orientation* (e.g., Wallace, 1956; Nuttin, 1964; Mehta et al., 1972) in the field of psychology. A positive future time orientation at school, according to research evidence, would result in an improvement in academic performance. In a similar vein, a person’s experience and manifestation of compassion is well-documented with other religious faiths, for example, Islam (i.e., the concept of *rahmah*) (Engineer, 2001; Taib, 2004; Nasir, 2016) and Christianity (Cornelius, 2013; Godlaski, 2015; Zylla, 2017).

### A Holistic Self: Spirituality, Enlightenment, and Connectedness

Holistic self, from our proposition, is a virtuous and magnanimous entity that arises from the study of life education. This consideration of a person’s holistic self is personal for us, drawing from our own teaching experiences, existing theoretical understanding, and ongoing research development. A person’s holistic self, we contend, reflects his/her state of liveliness, awakening, and spirituality, yielding a number of virtues and life qualities (e.g., indication of compassion) that largely support the paradigm of positive psychology (Seligman, 1999, 2010; Seligman and Csíkszentmihályi, 2000) in helping to address different types of pathologies and/or the achievement of optimal best. Let us consider a negative condition of death and how a person would overcome this fate. Death as a topic is dark, negative, and something that not many of us would like to discuss and/or talk about. Death is the ultimate destruction of life. For a person who is approaching death, it is a daunting and suffering experience. By the same token, for a close relative of someone who is dying, this “confrontation” of death is also a daunting experience, for example, the self-cognizance of grief, denial, and pain. How would positive psychology and, more importantly, a person’s holistic self-address this fundamental topic? Likewise, how would life education assist someone who is approaching death, and/or the close relative who is experiencing the onset of grief, etc.?

Referring to our previous discussion, an acquired spiritual and enlightened self (i.e., a person’s holistic self) in this case may provide relevant information (e.g., meaningful understanding of life wisdom) and experiences (e.g., realization that everything in life is aesthetic) (Loden, 1996; Phan et al., 2020a), which could assist a person to confront and cope with death. Buddhist faith, in particular, could encourage and instill the following comparable beliefs and/or perceptions:

i. The spiritual belief of samsāra, that is, the endless cycle of birth, rebirth, and redeath. That is, in this case, every rebirth is temporary and impermanent. Upon death, a person is reborn elsewhere in accordance with his/her own karma. Hence, from this spiritual belief, those who are approaching death may view death with a sense of encouragement, hope, and optimism, knowing that this is simply a cycle of life and death.
ii. The spiritual belief that the nature of life and death is aesthetic and that negativity, pain, suffering, etc. are part of the norm and subjective, depending on a person’s resolve, outlook, open-mindedness, etc. This emphasis connotes, in particular, the importance of enlightenment, which would potentially allow a person in this case to achieve the everlasting the beatitude of life.
ii. The spiritual belief into the esoteric nature of life and death, which may involve the possibility of transcendence beyond the realm of the physical world. In other words, spiritual cultivation may allow a person to view death as not being the ultimate end, but rather as a dividing line that separates one physical world life cycle from that of another cycle.

In contrast to death, pathologies, and other forms of maladaptive functioning, likewise, a person’s acquired holistic self is also able to facilitate the achievement of optimal best (Fraillon, 2004; Liem et al., 2012; Phan et al., 2020b) and the experience of flourishing (Diener et al., 2009; Seligman, 2010; Phan et al., 2019a). Our consideration in this matter connotes that virtues (e.g., compassion) and quality characteristics (e.g., having a positive outlook), in this case, may reflect a person’s optimal “individual experience.” In other words, a person’s achievement and/or experience of spirituality and enlightenment is more than just perceived evidence of positivity; rather, we rationalize that development of the holistic self is the ultimate optimal achievement or fulfillment that a person may experience. This optimal personal experience of spirituality and enlightenment, enabling a person to feel connected with God or some divine being (Nasr, 1987/2008; Bonab et al., 2013; Goswami, 2014; Van der Merwe, 2018), is of an exceptional level, which many of us may not achieve.

## Conclusion

The study of death education in Taiwan is extremely prominent. Over the past four decades, institutions have offered degree programs and courses that emphasize the importance of life education. One unique aspect of life education in Taiwan has been the incorporation of specific Eastern-derived epistemologies, philosophical and religious beliefs, and cultural practices. This uniqueness has led to the conceptualization and meaningful understanding that life education, in general, is concerned with enrichment of life qualities, cultivation of spiritual wisdom, and the pursuing of personal contentment and happiness. The main premise in this case is that aside from social stability and financial wealth, the notion of having a spiritual, fulfilling life is a noteworthy feat for development. Hence, on a daily basis, many Taiwanese engage in Buddhist meditation and other forms of meditation, as well as partaking in charitable acts and short courses, which would facilitate in the achievement and fulfillment of life qualities.

Our premise, as explored in the preceding sections, is to consider the extent to which life education, from the perspective of Taiwanese education, could coincide with and/or support the study of positive psychology (Seligman, 1999, 2010; Seligman and Csíkszentmihályi, 2000). This consideration is innovative as it emphasizes the potential nexus in terms of epistemologies, philosophical reasoning, and theoretical psychology between Western and Eastern contexts. The study of life education in Taiwan, for example, is interesting, delving into the complex nature of three interrelated elements that, in this sense, incorporate Eastern-derived epistemologies (e.g., Buddhist spirituality): life wisdom, life practice, and life care. Our concerted effort, in this analysis, led to the proposition of a theoretical–conceptual model for continuing research development. The proposed point of convergence, labeled as “X” in Figure 2, is significant, highlighting a person’s spiritual and enlightened self. This point of convergence, which we termed as spiritual wisdom and life enlightenment, is positive and, more importantly, would form part of a person’s holistic development (Forbes, 2003; Hare, 2010).

Overall, then, what is the main premise of our conceptual analysis article and, more importantly, our proposition? For us, as scholars, we have a collective interest to seek understanding into the proactivity of human agency—for example, does having a positive mindset assist a person to flourish, and/or to cope with an impending health problem? From the “Western” literature, we note that the paradigm of positive psychology (Seligman, 1999, 2010; Seligman and Csíkszentmihályi, 2000) has been effective in assisting individuals with their well-being experiences (e.g., facilitating positive emotional well-being, via means of resilience). Our viewpoint, largely derived from teaching and research development, likewise, considers the use of life education theories (Huang, 1993; Chen, 2013; Huang, 2014) to gauge into and facilitate the proactivity of human agency. In this case, we hope that researchers, educators, organizations, etc. will consider the use of life education and, in particular, our proposition (e.g., a person’s holistic self) to encourage and promote quality life experiences. Quality life experiences for a person, in this case, may entail the following: (i) to appreciate and value life, regardless of personal hardship, socioeconomic standing, obstacles, etc.; (ii) to seek meaningful daily understanding, insights, and experiences about life and to live a meaningful life; and (iii) to plan and to have positive future outlooks about life, including death and other life-related negativities.

Our intent, as scholars of education and psychology, is to seek innovative and new frontiers in theory and research development, which may then assist students and educators alike in their learning and teaching experiences. A focus on the seeking of life wisdom, spiritual cultivation (e.g., endeavor and/or perceived connectedness with some divine being), and experience of enlightenment (e.g., personal feeling of contentment and inner peace) is a noteworthy feat for consideration, especially in terms of applicability, practicality, and continuing research development. Foremost, from the present article, we encourage readers to capitalize on our research proposition of life education and to consider other inquiries for development. Our future goal, interestingly, is to use theoretical psychology and philosophical reasoning to unify comparable theories (e.g., life education, mindfulness, positive psychology) and cross-cultural viewpoints into an overarching framework for understanding—namely, to date, we have inquired into the process of optimization, the nature of holistic psychology, the multifaceted nature of mindfulness and the potential interrelationship between mindfulness and positive psychology, and of course life education and positive psychology. Of particular interest for us, in this case, is to develop and propose a unified theoretical model that could explain a person’s holistic development in terms of his/her subjective well-being (e.g., emotional well-being), cognition, morality, and social relationship. Is this feat of developing a unified model possible, and/or can we develop a theoretical model of human agency (e.g., a person’s achievement of optimal best in a particular domain of functioning) that could successfully take into account different philosophical beliefs, religious faiths, theoretical perspectives, etc.?

### Caveats and Future Directions

Theoretical psychology, philosophical reasoning, epistemologies, etc. are interesting “academic strategies” and/or approaches that we could use to develop new innovative theories and conceptualizations. Like any proposed theory or theoretical model for that matter, however, it is important that we are able to scientifically validate a particular line of inquiry, theoretical model, conceptual framework, etc. For example, in terms of quantitative research, it is perceived as being straightforward to investigate, say, a one-factor (e.g., Brown and Ryan, 2003; Chadwick et al., 2008), a two-factor (e.g., Cardaciotto et al., 2008; Davis et al., 2009), and/or a more complex factorial structure of mindfulness (e.g., a four-factor model: Baer et al., 2004; Feldman et al., 2007) using Likert-scale inventories with confirmatory factor analysis techniques (Kline, 2011; Byrne, 2012). In a similar vein, despite modest empirical development at present, it is possible to explore and empirically validate the nature of subjective well-being (Diener et al., 2009, 2010; Wiese et al., 2018).

We acknowledge that our discussion in this article is theoretical and that, at present, there is only limited empirical evidence (e.g., Figure 1) to support its premise. In this sense, it is not always feasible and/or achievable to validate an inquiry, especially when it is esoteric and non-scientific in nature. For example, in accordance with our previous discussion, we contend that it is somewhat unachievable to design an appropriate methodological design for usage, which could measure, assess, and validate different types of esoteric experiences (e.g., “tranquility,” “enlightenment,” “samsāra”). In this analysis, we are extremely constrained in our quest to validate the experience of and the concept of transcendence—that is, the possibility of life transcending beyond death. How would we scientifically determine whether this notion of postdeath experience is plausible?

One interesting topic and/or line of inquiry in the social sciences is related to methodological design (Bordens and Abbott, 2008; Gravetter and Forzano, 2009; Babbie, 2014), which a researcher could use to investigate and validate a particular concept, relationship, etc. For example, as a question for consideration, would a two-group experimental design (e.g., control group vs. experimental group) be the course for usage in terms of validating the negative impact of cognitive load imposition (Sweller et al., 2011; Sweller, 2012)? In our research development (Phan et al., 2020a) and, in particular, our recent conceptual-analysis article (Phan et al., 2019a), we introduced a term, coined as “methodological appropriateness.” Methodological appropriateness, in brief, is concerned with a researcher’s consideration of the appropriateness (or inappropriateness) and adequacy (or inadequacy) of a methodological design that he/she would use to validate a concept, association, etc. Methodological appropriateness, from our point of view, may explain limitations, as well as the ineffectiveness, inconsistency, and inaccuracy of established findings of empirical research. Poignant to this discussion, though, is the fact that the topic of methodological appropriateness (Phan et al., 2019a, 2020a) is also associated with conceptualizations and theorizations drawn from philosophical reasoning, theoretical psychology, and non-scientific intuitions. In other words, it is still plausible for researchers to consider the relevance and applicability of methodological appropriateness for esoteric and non-scientific matters.

From the above, we contend there are a number of caveats that are worthy for continuing research development. Foremost, in this analysis, is the “scientific” validation of our proposed conceptualization, as shown in Figure 2. With reference to Figure 2, there are two notable issues for researchers to consider:

i. Develop Likert-scale measures [e.g., (1) not true at all, (3) neutral, (5) complete true], other forms, which could assist in the measurement and assessment of positive life characteristics, such as a perceived sense of spirituality (e.g., Likert-scale rating of item: “I often experience a sense of spirituality for the unknown”), forgiveness (e.g., Likert-scale rating of item: “I am a forgiving person”), benevolence (e.g., Likert-scale rating of item: “I find it fulfilling to help others in the community”), etc.
ii. Develop a comparable framework, which we could perhaps use to cross-validate the described theoretical model as depicted in Figure 2. Researchers often use this methodological approach to cross-validate and establish psychometric properties of a Likert-scale measure. The rationale, in this case, is that comparable measures would positively associate with each other [e.g., Measure A ↔ Measure B, where ↔ (i.e., association) is postulated to be positive] and that comparable measures would exert similar predict effects on an outcome [e.g., Measure A → O (β_A_ effect), Measure B → O (β_B_ effect), where β_A_∼β_B_].

In a similar vein, as one of our reviewers recently pointed out, our conceptualization of the spiritual and enlightened self may have relevance and applicability to the Western context. In other words, in addition to Buddhist teaching (Yeshe and Rinpoche, 1976; Sheng Yen, 2010), it is plausible for us to consider the impact of other cultural and religious faiths, which could also initiate, instill, and facilitate a person’s spiritual belief regarding life [e.g., Christianity (Davis, 2004; Knitter, 2009; Van der Merwe, 2018), Hinduism (Warrier, 2006; Srivastava and Barmola, 2013; Goswami, 2014), Islam (Nasr, 1987/2008; Bonab et al., 2013; Marzband et al., 2017), etc.]. In a similar vein, resonating with the focus of our conceptual analysis, we contend that support for the study of positive psychology (Gilham and Seligman, 1999; Seligman, 1999; Seligman and Csíkszentmihályi, 2000) could involve other cultural practices, religious faiths, and theoretical understanding. An important question then for consideration is whether and/or to what extent there is a central point of commonality between different cultural practices, religious faiths, and theoretical understanding, which in turn could support the paradigm of positive psychology and/or the teaching of life education. In this sense, is it plausible for us to establish some form of “convergence” in terms of commonalities of theoretical understanding between, say, Christianity, Buddhism, and Islam? How does this convergence point, in particular, assist a person to appreciate, value, and/or understand the true meaning of life? By all accounts, we contend the possibility that there are contrasting and diverse viewpoints, resulting in dissimilarity and inconsistency with reference to the study of life education. For example, in terms of life wisdom, we note that Hinduism also places emphasis on the notion of *moksha*, or the freeing of the samsāric cycle altogether (Warrier, 2006). Spiritual enlightenment, the personal striving for inner discipline, and one’s detachment from the external world at large may all assist a person to free himself/herself from the endless cycle of birth, death, and rebirth.

Social sciences research, as we mentioned, places emphasis on the topic of methodological appropriateness and the use of different methodological data collection techniques to gather evidence. Some subject matters are relatively straightforward, relying on quantitative methodological approaches and robust statistical analyses. It is sound and possible, from our point of view, for researchers to consider their own teaching practices, professional and personal experiences, personal reflections and interpretations of life, and social interactions as “anecdotal evidence,” which could provide insights and theoretical understanding into the study of life education. For example, documenting our own experiences of meditation and teaching of mindfulness (Hanh, 1976; Chen, 2012; Phan et al., 2020a) has helped us to appreciate the importance of spirituality and the meaning of life wisdom as opposed to cognitive intelligence in a subject matter. Continuing practice of Buddhist meditation, in this sense, has encouraged and motivated us to engage in benevolent acts. By the same token, our anecdotal experiences, in tandem with our existing research and personal intuitions, have enabled us to develop different conceptualizations and theorizations of positive psychology (e.g., the theory of optimization) (Phan et al., 2017, 2019a). Researchers may adopt our lead and take a similar pathway, sharing with the academic community personal practices, experiences (e.g., esoteric experience), intuitions, etc. that could, likewise, assist in theoretical understanding of life education.

Having said this, however, we acknowledge that personal anecdotal evidence is non-scientific and contentious in nature, raising the question of validity, acceptance, and generalization. To a certain degree, the same point of acknowledgment also lends itself to the study of positive psychology, which has received relatively modest scientific evidence to date. On this basis, we encourage researchers to consider pathways, conceptualizations, methodological designs, etc. that could, similarly, help validate the paradigm of positive psychology. In this analysis, taking our cue, it is plausible to consider the establishment of “proxy” evidence that could affirm the prevalence of positive psychology.

## Author Contributions

HP and BN were responsible for the literature search and write-up of this article. HP, BN, SC, LW, WL, and CH contributed equally to the conceptualization and theoretical contribution of the article.

## Conflict of Interest

The authors declare that the research was conducted in the absence of any commercial or financial relationships that could be construed as a potential conflict of interest.
